# Association of composite dietary antioxidant index and peripheral artery disease: a national cross-sectional study

**DOI:** 10.3389/fnut.2025.1606769

**Published:** 2025-09-10

**Authors:** Zhi Fan, Rongrong Zhu, Shuang Guo, Qi Wu, Jiazheng Li, Shibiao Liu, Zhixiang Su, Weiwei Wu

**Affiliations:** Department of Vascular Surgery, Beijing Tsinghua Changgung Hospital, School of Medicine, Tsinghua University, Beijing, China

**Keywords:** oxidative stress, antioxidant, CDAI, PAD, NHANES

## Abstract

The Composite Dietary Antioxidant Index (CDAI), a comprehensive measure of dietary antioxidant intake, quantifies the combined effects of key micronutrients, including vitamins A, C, and E, zinc (Zn), and selenium (Se), to evaluate overall antioxidant capacity. Existing evidence suggests that CDAI is inversely associated with cardiovascular diseases, including myocardial infarction and stroke. This study aims to investigate the relationship between CDAI and peripheral artery disease (PAD), which remains unclear in the current literature. In this study, we analyzed data from 2,332 participants with available ankle-brachial index (ABI) measurements from the NHANES database. Multivariable logistic regression and smooth curve fitting were employed to evaluate the association between CDAI and PAD. Additionally, subgroup analyses and interaction tests were conducted to assess the generalizability and stability of these relationships. Our findings revealed a significant inverse association between CDAI and PAD. In the fully adjusted model, each one-unit increase in CDAI was associated with a 12% reduction in PAD prevalence (OR = 0.88, 95% CI: 0.81–0.95). Moreover, participants in the highest quartile of CDAI had a 53% lower likelihood of developing PAD (OR = 0.47, 95% CI: 0.24–0.93) compared with those in the lowest quartile. These results demonstrate a strong correlation between CDAI and PAD risk, suggesting that diets rich in antioxidants (reflected by higher CDAI scores) may play a role in PAD prevention. However, further comprehensive research and prospective cohort studies are needed to explore causal relationships and validate these findings.

## 1 Introduction

PAD, the manifestation of atherosclerosis in lower extremity arteries, is a common cardiovascular condition linked to increased risks of amputation, myocardial infarction, stroke, and death (1). PAD is an established predictor of all-cause mortality and cardiovascular death. Current epidemiological evidence demonstrates that PAD significantly raises the risk of all-cause mortality, cardiovascular death, coronary artery disease, and cerebrovascular disease (2). The global burden of PAD affects more than 200 million people, corresponding to over 3% of the world’s population (3).

The CDAI developed by Wright et al. (4) is an integrative scoring system that measures cumulative antioxidant intake (including vitamins A, C, E, selenium, zinc, and carotenoids) from dietary sources. A higher CDAI has been associated with a reduced risk of multiple chronic conditions, including diabetes, chronic obstructive pulmonary disease (COPD), low back pain, and sleep disorders (5–8). Two additional studies have, respectively, identified inverse relationships between the CDAI and the probability of cardiovascular disease in general adult populations and postmenopausal women (9, 10).

However, no studies have investigated the association between CDAI and the prevalence of PAD. Therefore, we initiated cross-sectional research to explore the relationship between CDAI and PAD using data from the 1999–2004 NHANES.

## 2 Materials and methods

### 2.1 Study population

**Figure 1:**
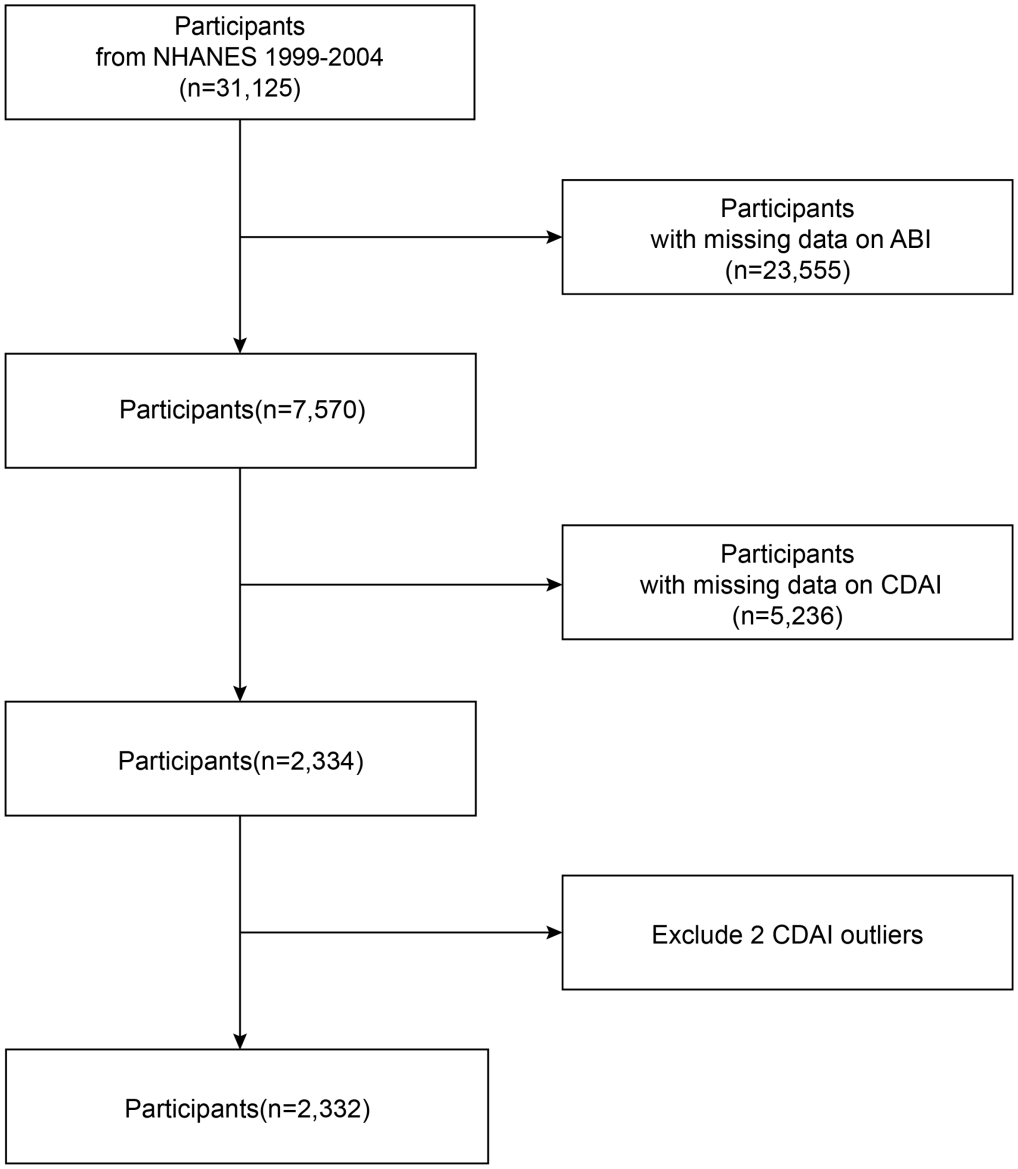
A detailed flow chart of participant recruitment.

NHANES is an extensive health assessment initiative conducted by the Centers for Disease Control and Prevention (CDC). The research design and associated materials obtained formal authorization from the designated regulatory authorities and were approved through ethical review. Because NHANES data on the ABI questionnaire were only available from 1999 through 2004, our study primarily focused on this period, and 31,125 people were included in this research. The exclusion criteria were as follows: (a) no ABI data (23,555); (b) no CDAI data (5,236); and (c) CDAI outliers (2). Based on the above criteria, 2,332 participants were enrolled. The baseline information of the participants is illustrated in Figure 1.

### 2.2 Composite dietary antioxidant index

CDAI served as the primary exposure factor in this study. It was derived from the NHANES Dietary Interview Questionnaire and calculated using a specific formula. Existing literature has validated the reliability of this methodological approach (11–13). The questionnaire collects dietary intake data over any two consecutive days within a calendar year, and calculates the intake of various nutrients and trace elements. The data were collected using two different methods: the first time at the Mobile Examination Center (MEC), a specially designed bus, and the second time via telephone follow-up. The specific dietary intake collection items and the proportion of nutritional components are defined by the USDA’s (U. S. Department of Agriculture) Dietary Research Food and Nutrition Database (FNDDS). To calculate CDAI, we selected the daily intake of six dietary antioxidants (vitamins A, C, E, selenium, zinc, and carotenoids) obtained from the Dietary Interview Questionnaire (Days 1 and 2). The CDAI is computed as the sum of standardized values for each antioxidant using the formula:


$$\text{CDAI}=\sum_{i=1}^{6}\frac{\text{each intake}-\text{mean}}{\mathrm{SD}}$$

### 2.3 Peripheral artery disease

We evaluated the ankle-brachial blood pressure index (ABI) as an indicator for assessing lower extremity disease using the NHANES examination data component. During the measurement, participants were required to lie on the examination table in the supine position. A cuff tonometer was placed on the right arm and both ankles, and blood pressure was measured at each site. If the subject’s right upper extremity had skin lesions, amputation, or contraindications to compression, the left arm was used. According to the protocol, measurements were taken twice at each site, while for participants older than 65 years, only one measurement was taken. Finally, the ABI was calculated by the ratio of ankle systolic blood pressure to brachial systolic blood pressure. Based on clinical guidelines, participants with an ABI ≤ 0.9 in either leg were defined as suffering PAD (14).

### 2.4 Covariables

In this study, covariates were rigorously selected based on two principles: (1) documented associations with PAD in previous research, or (2) recognition as established clinical risk factors supported by epidemiological studies or consensus guidelines. The included covariates encompassed demographic characteristics (age, gender, race), socioeconomic factors (education level, marital status, income-to-poverty ratio), anthropometric and lifestyle measures—including body mass index, physical activity levels, dietary factors (excluding antioxidants such as total energy, calcium, and iron intake), and adverse health behaviors (smoking status and alcohol consumption). Additionally, comorbid conditions (diabetes mellitus, hypertension, and hypercholesterolemia) were incorporated due to their well-established role in PAD pathogenesis, pro-inflammatory and pro-oxidative effects, and potential confounding influence on the relationship between CDAI and PAD outcomes. Adjustment for these covariates enhances the robustness of our analyses by minimizing residual confounding, thereby improving the validity and interpretability of the observed associations.

### 2.5 Statistical analysis

The baseline characteristics of the participants were evaluated using the chi-square test and t-test. The effect sizes of exposure factors on outcomes were examined using logistic regression analyses. In this study, three distinct multivariate logistic regression models were employed to investigate the relationship between CDAI and PAD, while progressively adjusting for and controlling potential confounders to more accurately assess the independent associations between the two variables. These models differed in their covariate adjustments: Model 1 was unadjusted for any covariates, while Model 2 included age, gender, race, and educational status. In contrast, Model 3 combined covariates including gender, age, race, marital status, education level, household poverty rate, BMI, physical activity, energy intake, calcium intake, iron intake, drinking status, smoking status, diabetes, hypertension, and high cholesterol. Additionally, CDAI was divided into quartiles of continuous measurements to allow for trend analysis to determine potential correlations with PAD, with values categorized as follows: quartile 1 (Q1): −5.62 to −2.26; quartile 2 (Q2): −2.25 to −0.75; quartile 3 (Q3): −0.74 to 1.18; and quartile 4 (Q4): 1.19 to 29.17. Smoothed curve fitting methods were used to evaluate nonlinear correlations. Stratified multiple regression analysis with interaction terms was conducted to assess heterogeneity and stability across subgroups, including sex, age, race, education, PIR, BMI, drinking, smoking, diabetes, hypertension, and hypercholesterolemia. All statistical analyses were conducted utilizing R software (version 3.4.3) in conjunction with EmpowerStats (version 2.0); statistical significance was defined as *p* < 0.05.

## 3 Results

### 3.1 Sociodemographic and clinical characteristics

Baseline sociodemographic and clinical characteristics of the participants are presented in Table 1. A total of 2,332 participants aged over 40 years were included in this analysis. The mean age was 60.27 years (SD = 12.74). Among the participants, 50.21% were male, and 47.60% identified as non-Hispanic White. The mean CDAI score for the overall cohort was 0.02 (SD = 3.46). Participants with PAD had significantly lower CDAI values than those without PAD (−1.08 vs. 0.06, *p* < 0.001). 7.12% of participants were diagnosed with PAD. Participants with PAD were generally older and more likely to be non-Hispanic Black, smokers, or have hypertension, diabetes mellitus, or hypercholesterolemia. They were also more likely to have family PIR ≤ 100% and lower energy intake. In addition, patients with PAD had lower educational levels. Significant differences appeared in marital status, with higher proportions of widowed/divorced/separated PAD participants and lower proportions of married/living with a partner. Individuals with PAD tended to have a BMI in the normal or overweight range.

**Table 1:** Clinical characteristics of study population.

Variables	Overall	ABI >0.9	ABI ≤0.9	*p*-value
N, %	2,332(100%)	2,166(92.88%)	166(7.12%)	**<0.001**
Age (years)	60.27 ± 12.74	59.49 ± 12.50	70.49 ± 11.29	**<0.001**
Gender, %				0.670
Male	1,171 (50.21%)	1,085 (50.09%)	86 (51.81%)	
Female	1,161 (49.79%)	1,081 (49.91%)	80 (48.19%)	
Race/Ethnicity, %				**0.008**
Mexican American	628 (26.93%)	596 (27.52%)	32 (19.28%)	
Other Hispanic	125 (5.36%)	118 (5.45%)	7 (4.22%)	
Non-Hispanic White	1,110 (47.60%)	1,026 (47.37%)	84 (50.60%)	
Non-Hispanic Black	408 (17.50%)	366 (16.90%)	42 (25.30%)	
Other	61 (2.62%)	60 (2.77%)	1 (0.60%)	
Education level, %				**0.002**
<9th grade	541 (23.20%)	492 (22.71%)	49 (29.52%)	
9–11th grade	445 (19.08%)	407 (18.79%)	38 (22.89%)	
High school graduate	513 (22.00%)	469 (21.65%)	44 (26.51%)	
Some college or AA degree	461 (19.77%)	440 (20.31%)	21 (12.65%)	
College graduate or above	372 (15.95%)	358 (16.53%)	14 (8.43%)	
Family PIR, %				**0.029**
≤100	367 (15.74%)	331 (15.28%)	36 (21.69%)	
>100	1965 (84.26%)	1835 (84.72%)	130 (78.31%)	
BMI (kg/m^2^), %				**0.004**
<18.5	25 (1.07%)	23 (1.06%)	2 (1.20%)	
18.5–24.9	624 (26.76%)	565 (26.08%)	59 (35.54%)	
25.0–29.9	916 (39.28%)	846 (39.06%)	70 (42.17%)	
≥30.0	767 (32.89%)	732 (33.80%)	35 (21.08%)	
Physical activity, %				0.714
Active	1962 (84.13%)	1824 (84.21%)	138 (83.13%)	
Moderate	370 (15.87%)	342 (15.79%)	28 (16.87%)	
Smoking, %				**<0.001**
Yes	1,238 (53.09%)	1,122 (51.80%)	116 (69.88%)	
No	1,094 (46.91%)	1,044 (48.20%)	50 (30.12%)	
Drinking, %				0.410
Yes	1,543 (66.17%)	1,438 (66.39%)	105 (63.25%)	
No	789 (33.83%)	728 (33.61%)	61 (36.75%)	
Diabetes, %				**<0.001**
Yes	298 (12.78%)	260 (12.00%)	38 (22.89%)	
No	2034 (87.22%)	1906 (88.00%)	128 (77.11%)	
Hypertension, %				**<0.001**
Yes	930 (39.88%)	837 (38.64%)	93 (56.02%)	
No	1,402 (60.12%)	1,329 (61.36%)	73 (43.98%)	
Hypercholesterolemia, %				**0.009**
Yes	796 (34.13%)	724 (33.43%)	72 (43.37%)	
No	1,536 (65.87%)	1,442 (66.57%)	94 (56.63%)	
CDAI	0.02 ± 3.46	0.06 ± 3.50	−1.08 ± 2.67	**<0.001**
Energy Intake (kcal)	1923.99 ± 888.95	1944.31 ± 901.60	1658.80 ± 649.33	**<0.001**
Calcium Intake (mg)	748.20 ± 519.36	753.80 ± 522.61	675.16 ± 470.26	0.060
Iron Intake (mg)	14.62 ± 9.24	14.73 ± 9.21	13.32 ± 9.52	0.059

Continuous data were presented as the mean and 95% confidence interval, category data were presented as the proportion and 95% confidence interval, CDAI, composite dietary antioxidant index, BMI, body mass index, PIR, poverty income ratio. Bold values indicate statistical significance (*p* < 0.05).

### 3.2 Association between CDAI and PAD

**Figure 2:**
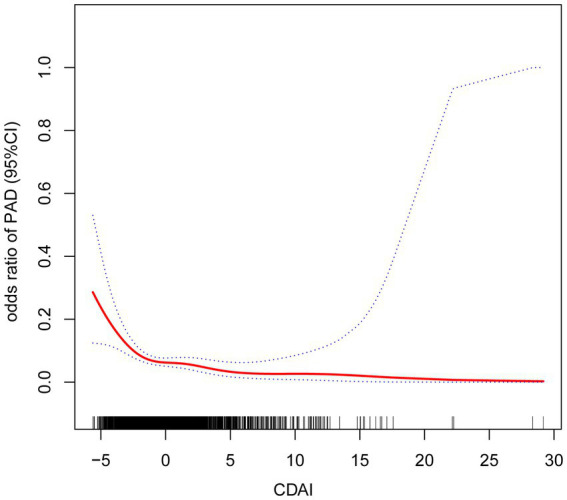
Results from smooth curve fitting.

The association between CDAI and PAD was assessed using logistic regression models. The findings from logistic regression on PAD are summarized in Table 2. After adjusting for all covariates, a one-unit increment in CDAI was linked to a 14% reduction in PAD prevalence [OR = 0.86 (95% CI: 0.78–0.95)]. After categorizing the CDAI into four groups (Q1-4) based on its values, the inverse association remained statistically significant (*p* < 0.05). Compared to participants in Q1 (reference group), those in Q4 had a 53% reduction in PAD risk [OR = 0.47 (95% CI: 0.24–0.93)]. The smoothed curve fitting visualized the relationship between CDAI and PAD, confirming a negative correlation between the two variates (Figure 2). The P for trend indicated a significant dose–response relationship (unadjusted model: *p* = 0.001; model I: *p* = 0.005; model II: *p* = 0.025). Threshold effect analysis also revealed a significant nonlinear relationship between CDAI and PAD prevalence (p for nonlinearity = 0.008). Using a two-piecewise linear regression model, an inflection point was identified at CDAI = −1.32. This result demonstrated distinct dose–response patterns on either side of this threshold. Below this value, each unit increase in CDAI corresponded to a 35% reduction in PAD probability (adjusted OR = 0.65, 95% CI: 0.51–0.82, *p* < 0.001). However, beyond this threshold, the protective association plateaued (adjusted OR = 0.92, 95% CI: 0.83–1.02, *p* = 0.097). No further clinical benefit was seen for increasing CDAI levels above this point (Table 3).

**Table 2:** Results from logistic regression analysis on PAD.

Result	Non-adjusted model		Model I		Model II	
	OR [95% CI]	*p* value	OR [95% CI]	*P* value	OR [95% CI]	*P* value
Continuous CDAI	0.87 (0.82, 0.93)	<0.0001	0.89 (0.83, 0.95)	0.0011	0.86 (0.78, 0.95)	0.0030
CDAI-Q1 (−5.62 to −2.26)	1 (ref)	—	1 (ref)	—	1 (ref)	—
CDAI-Q2 (−2.25 to −0.75)	0.69 (0.46, 1.05)	0.0806	0.63 (0.41, 0.98)	0.0411	0.57 (0.35, 0.92)	0.0203
CDAI-Q3 (−0.74 to 1.18)	0.57 (0.37, 0.88)	0.0114	0.55 (0.35, 0.87)	0.0110	0.52 (0.30, 0.91)	0.0212
CDAI-Q4 (1.19 to 29.17)	0.42 (0.26, 0.68)	0.0003	0.51 (0.31, 0.84)	0.0090	0.47 (0.24, 0.93)	0.0303
*P* for trend	<0.001		0.005		0.0025	

Model 1: no covariates were adjusted. Model 2: adjusted for age, sex, race/ethnicity, and education levels. Model 3: adjusted for age, sex, race/ethnicity, education levels, PIR, BMI, physical activity, energy intake, calcium intake, iron intake, smoking, drinking, hypertension, diabetes and hypercholesterolemia.

**Table 3:** Threshold effect analysis of CDAI on PAD using a two-piecewise linear regression model.

Inflection point	Adjusted OR (95%CI)	*P*-value
Model I		
One line effect	0.86 (0.78, 0.95)	0.0032
Model II		
Turning point (k)	−1.32	
< K effect 1	0.65 (0.51, 0.82)	0.0002
> K effect 2	0.92 (0.83, 1.02)	0.0968
Effect 2–1	1.42 (1.10, 1.83)	0.0073
Model fit value at K	−2.60 (−2.86, −2.35)	
Log-likelihood ratio		0.0080

### 3.3 Subgroup analysis

**Figure 3:**
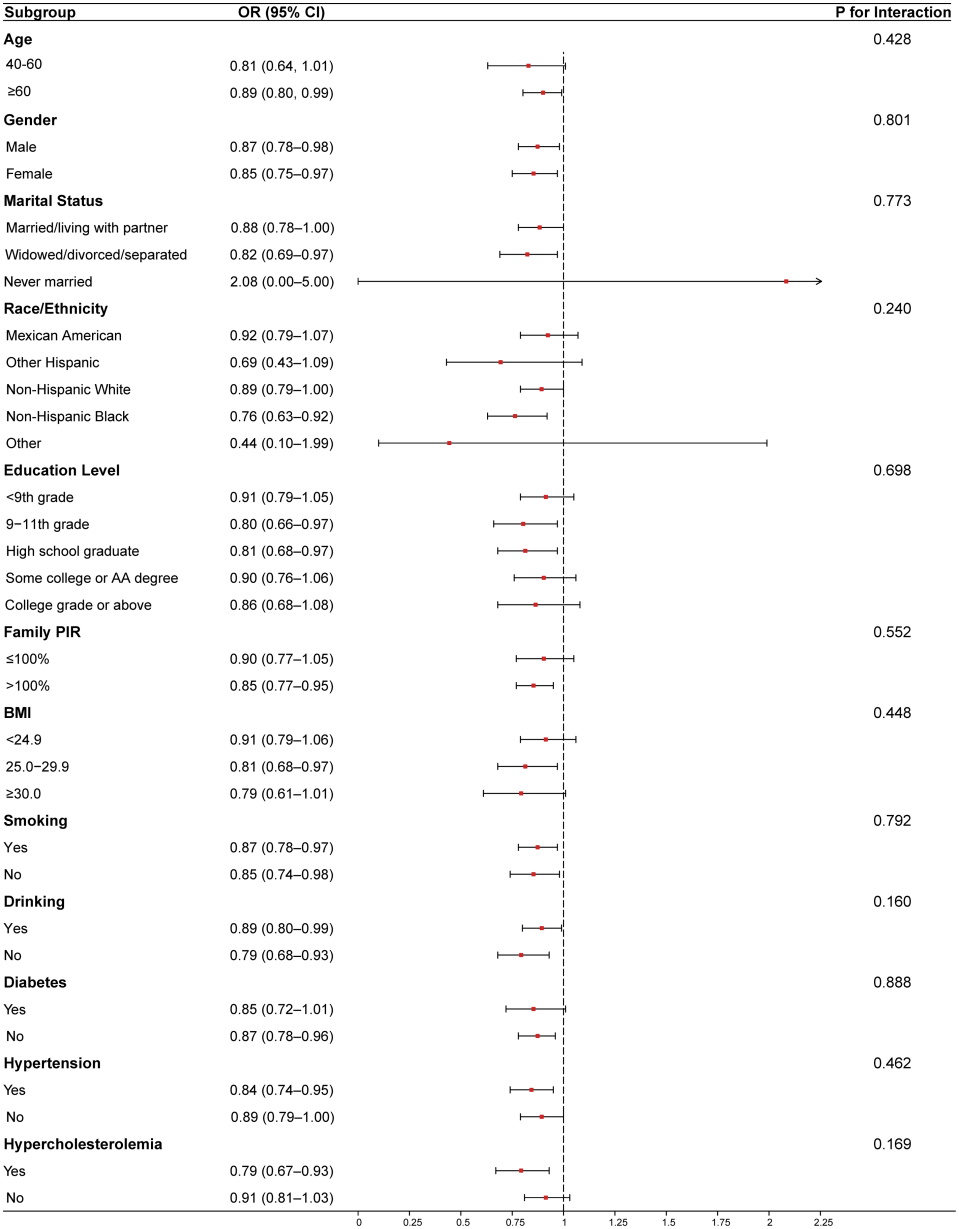
Results from subgroup analyses. Age, sex, race/ethnicity, education levels, PIR, BMI, physical activity, energy intake, calcium intake, iron intake, smoking, drinking, hypertension, diabetes and hypercholesterolemia were adjusted. Abbreviation: CDAI, composite dietary antioxidant index, BMI, body mass index, PIR, poverty Income Ratio.

**Figure 4:**
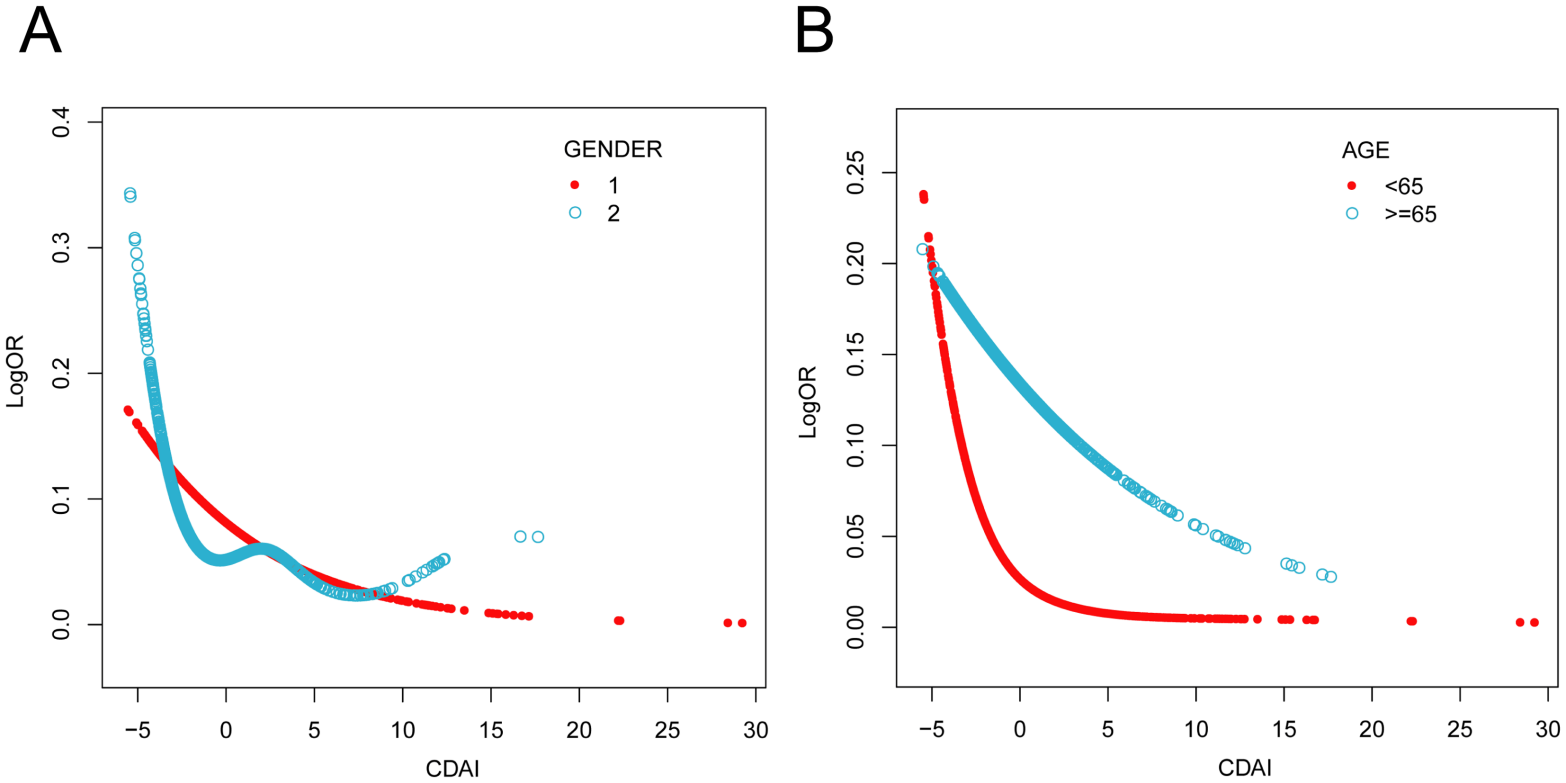
Results from subgroups smooth curve fitting analyses. **(A)** sex (male and female), **(B)** age (< 65 years, ≥ 65 years). Age, sex, race/ethnicity, education levels, PIR, BMI, physical activity, energy intake, calcium intake, iron intake, smoking, drinking, hypertension, diabetes and hypercholesterolemia were adjusted.

Analyses were stratified by sex, age, race, education, PIR, BMI, energy intake, calcium intake, iron intake, smoking, drinking, hypertension, diabetes, and hypercholesterolemia. The forest plot (Figure 3) shows a consistent and statistically significant inverse association between CDAI and PAD across all genders, age ≥65 years, Non-Hispanic Black, participants with family PIR above 100, 9th grade to high school graduates, widowed/divorced/separated participants, BMI 25.0–29.9, smokers, non-smokers, drinkers, non-drinkers, participants with hypertension, participants without diabetes, and participants with hypercholesterolemia. In all subgroup analyses, the interaction tests were statistically insignificant (*P* for interaction > 0.05), indicating the negative association has good robustness and generalizability. Smoothing curves (Figure 4) suggested steeper negative slopes in females and participants aged <65 years. However, formal interaction tests remained non-significant. The underlying mechanisms warrant further investigation.

## 4 Discussion

In the cross-sectional study, we enrolled 2,332 representative participants and observed the negative relationship between CDAI and PAD. This suggests that higher levels of dietary antioxidants may be linked to a lower risk of developing PAD, particularly in high-risk populations with antioxidant deficiency.

Our results suggest that CDAI may have potential clinical value in the prevention of PAD. To our knowledge, this is the first population-based study to evaluate the association between CDAI and PAD, independent of traditional cardiovascular risk factors. By analyzing the NHANES database, Teng et al. (11) demonstrated a negative association between CDAI and stroke, with the antioxidant-based nomogram model showing strong predictive capability for stroke risk. Analogously, Ma et al. (12) demonstrated that the CDAI exhibited an L-shaped inverse association with the risk of coronary heart disease (CHD), reducing it by up to 65%. A study has also found that CDAI improves Atherosclerosis, Maugeri et al. (15) using the Kardiovize Brno 2030 study found that CDAI reduced carotid intima-media thickness (cIMT) in women. Consistent with the results of the previous study, CDAI is a protective factor against cardiovascular diseases. In this study, we found a negative relationship between the CDAI and PAD, indicating that individuals with high levels of dietary antioxidants may have a lower risk of developing PAD.

Atherosclerosis is a major cause of stenosis or occlusion of the arteries lumen in the lower extremities, affecting the blood supply to the lower extremities. Oxidative stress, inflammatory response, and lipid infiltration are the primary causes of atherosclerosis formation (16). Of these, reactive oxygen species (ROS), the primary byproducts of oxidative stress, initiate atherosclerosis by inducing endothelial dysfunction, chronic inflammation, and dysregulated lipid metabolism (17). Moreover, oxidative stress critically influences PAD progression by promoting vascular inflammation and endothelial dysfunction (18, 19). Oxidative stress results from the overproduction of reactive oxygen species (ROS) combined with impaired or inadequate antioxidant defense systems (20, 21). Animal and clinical studies indicate that oxidative stress drives the initiation and progression of PAD via diverse mechanisms. In animal models, experimental hindlimb ischemia (HLI) studies have shown that the generation of ROS is closely associated with angiogenesis, endothelial dysfunction, and inflammatory responses (22). Increased muscle ROS formation was found after ligation of unilateral iliac and femoral arteries (23). Moreover, ROS interferes with vascular endothelial growth factor (VEGF) mediated angiogenesis by decreasing nitric oxide (NO) bioavailability, thereby affecting the recovery of ischemic tissues (24). In addition, animal studies of cigarette smoke exposure have demonstrated that major risk factors for PAD, such as smoking and diabetes, further exacerbate inflammation by enhancing oxidative stress and decreasing NO bioactivity (25, 26). In terms of clinical studies, a cross-sectional analysis comparing blood markers in patients with type 2 diabetes with or without PAD found higher levels of AGEs and MDA (two biomarkers closely linked to oxidative stress), and lower levels of vitamin E and total reactive antioxidant potentials (TRAPs) in patients with PAD compared to those without PAD (27). A small clinical study (*n* = 74,12 months duration) noted that propionyl L-carnitine improved ABI, maximum walking distance, pre-morbid walking distance, and quality of life in patients (28).

The key antioxidants included in the CDAI—such as carotenoids, vitamins A, C, E, and essential trace metals (e.g., Zn, Se)—exert protective effects against oxidative stress through diverse mechanisms. Vitamin C is a water-soluble compound generated through glucose metabolism (29, 30). It synthesizes the reducing agents needed for collagen fibers, and it also protects the body from free radicals. Moreover, Vitamin C has been demonstrated to ameliorate oxidative stress through the modulation of key signaling pathways (31). Carotenoids are structurally similar to vitamin A (32). They may be involved in antioxidant protection against oxidative free radical attack (33). Some studies indicate that vitamin A and carotenoids are potent antioxidants that may inhibit the development of cardiovascular diseases and enhance visual health (34, 35). They can impact both free radicals and peroxyl radicals. Vitamin E, as an essential nutrient that can only be ingested exogenously, is a fat-soluble vitamin group present in high concentrations throughout the body, including cell membranes and cytoplasmic proteins, and is involved in regulating the body’s redox balance (36). Numerous studies have demonstrated that Vitamin E exhibits anti-atherosclerotic and anti-cardiovascular effects (37, 38). The trace element selenium (Se) is regarded as a beneficial dietary supplement due to its significant antioxidant capabilities and function of enhancing health (39). Amino acids, peptides, and enzymes are selenium-containing compounds, also playing crucial biological roles in the human body (40). Selenium and its derivatives, especially selenoproteins containing selenocysteine form selenium, have been shown to have antioxidant properties (41). Zn(II), Cu(I), and iron, the metal thiolate groups, also function as redox switches (42), serving as crucial and irreplaceable elements in the reaction pathway.

However, the US Preventive Services Task Force (USPSTF) found through a systematic review of RCTs that current evidence is insufficient to evaluate the balance of benefits and risks of using single or combined nutritional supplements for the prevention of certain diseases (43). Findings from retrospective research also support similar conclusions (44, 45).

Although the effects of single nutrients are not significant or insufficiently supported by evidence, some studies have found that the Mediterranean diet, rich in antioxidant components, is linked to a lower risk of cardiovascular disease and PAD (46). The Mediterranean Diet is predominantly plant-based, particularly in fruits, vegetables, legumes, and nuts, which are abundant in antioxidants such as vitamin A, C, E, polyphenols and others (47). The Mediterranean diet has been used for the prevention of cardiovascular diseases, including peripheral arterial disease and ischemic stroke. The Mediterranean diet was found to be superior to a low-fat diet in the prevention of cardiovascular events, including peripheral arterial disease, myocardial infarction, and ischemic stroke, in a 1,000-person RCT, supporting the secondary prevention of cardiovascular disease through the Mediterranean diet (48). Additionally, an exploratory, non-prespecified analysis of a large-scale randomized controlled trial revealed that the Mediterranean diet, characterized by an unrestricted energy intake and rich in antioxidants, significantly reduces the prevalence of PAD, underscoring its value in cardiovascular disease prevention (49, 50).

Interestingly, as depicted in Figure 4, our study revealed a potential differential benefit of antioxidant dietary intake across sex and age groups. Specifically, female participants and individuals under 65 years of age exhibited a more pronounced reduction in PAD risk with increasing CDAI levels. Accumulating evidence indicates that female sex hormones, particularly estrogen, exert multifaceted atheroprotective effects (51). A prospective study on oxidative balance scores and hypertension also observed similar sex-specific differences and demonstrated that it may be mediated by oxidative stress mechanisms (52). In elderly individuals, advanced age drives vascular aging process. It includes oxidative stress, endothelial dysfunction, and progressive arterial stiffening, which collectively may compromise the efficacy of antioxidant interventions (53). Therefore, this may raise different guidelines for the prevention of PAD in people of different genders and ages. For instance, premenopausal women or middle-aged people at risk of PAD might benefit from a diet plan that emphasizes foods high in antioxidants, while older populations may require adjunct therapies to overcome age-related declines in antioxidant absorption or utilization.

This study has several advantages. Firstly, this study is grounded in data extracted from the NHANES, which employs a randomized and scientifically rigorous sampling methodology. This approach ensures that the selected cohort reflects the demographic diversity of the US population, providing a robust representation of the US population’s health and nutritional status. Additionally, this study benefits from a substantial sample size, enhancing statistical precision and reliability. Additionally, we also adjusted for a large number of covariates to make the results more independent and more credible. Nevertheless, the limitations of this study must be acknowledged. Firstly, due to the cross-sectional study design, the authors were unable to establish clear causal relationships. Secondly, dietary information was self-reported, which may introduce inaccuracies due to recall bias. Moreover, due to the NHANES study protocol, which excluded individuals aged below 40 years from screening for these variables, the association between CDAI and PAD could not be comprehensively evaluated across a broader demographic spectrum.

## 5 Conclusion

In conclusion, our study demonstrates that CDAI is inversely associated with PAD in U. S. adults aged ≥40 years. Moreover, this association remained consistent across all subgroups, supporting the potential role of antioxidant-rich diets (reflected by higher CDAI scores) in PAD prevention in the general population. However, further comprehensive research and prospective cohort studies are needed to explore causal relationships and validate these findings.

## Data Availability

Publicly available datasets were analyzed in this study. This data can be found at: wwwn.cdc.gov/nchs/nhanes/.
